# Case Report: When dual immune checkpoint blockade strikes back: cadonilimab-induced hypersensitivity in solid tumors — a case series and review

**DOI:** 10.3389/fimmu.2025.1643279

**Published:** 2025-08-11

**Authors:** Ping Song, Yuqi Jin, Linglin Fu, Fengming Yang, Yinuo Tan

**Affiliations:** ^1^ Department of Nursing, The Second Affiliated Hospital, Zhejiang University School of Medicine, Hangzhou, Zhejiang, China; ^2^ Department of Medical Oncology, Key Laboratory of Cancer Prevention and Intervention, Ministry of Education, The Second Affiliated Hospital, Zhejiang University School of Medicine, Hangzhou, Zhejiang, China; ^3^ Zhejiang Provincial Clinical Research Center for Cancer, Hangzhou, China; ^4^ Cancer Center of Zhejiang University, Hangzhou, China; ^5^ Center for Medical Research and Innovation in Digestive System Tumors, Ministry of Education, Hangzhou, China; ^6^ School of Renji Medical Sciences, Wenzhou Medical University, Wenzhou, China

**Keywords:** cadonilimab, hypersensitivity reaction, infusion-related reaction, immune checkpoint inhibitor, case report, literature review

## Abstract

**Background:**

Immune checkpoint inhibitors (ICIs) have revolutionized cancer therapy, but immune-related hypersensitivity reactions remain a clinical concern. Cadonilimab, a novel PD-1/CTLA-4 bispecific antibody, has demonstrated encouraging antitumor efficacy across various solid tumors; however, hypersensitivity or infusion-related reactions may occasionally occur.

**Methods:**

We herein report five cases of cadonilimab-induced allergic or infusion-related reactions in patients with different advanced solid tumors. Clinical manifestations ranged from mild skin rash to severe anaphylaxis with hypotension. All patients were managed promptly with individualized anti-allergic interventions, and some were able to safely continue therapy with modified infusion protocols.

**Results:**

The series emphasizes the importance of early identification and tailored management of hypersensitivity reactions during cadonilimab treatment. Additionally, a comprehensive literature review was conducted summarizing current clinical trials, case reports, and real-world evidence regarding cadonilimab’s efficacy and safety across multiple cancer types.

**Conclusion:**

Our findings highlight both the potential risks and manageable nature of cadonilimab-induced hypersensitivity, supporting its continued clinical application with appropriate monitoring and management strategies.

## Introduction

Immune checkpoint inhibitors (ICIs) targeting the PD-1/PD-L1 and CTLA-4 pathways have dramatically improved clinical outcomes across a variety of malignancies (1–4). Cadonilimab (AK104), a novel bispecific antibody simultaneously targeting PD-1 and CTLA-4, has received regulatory approval in China for the treatment of several advanced solid tumors (5). While cadonilimab is generally well tolerated, infusion-related hypersensitivity or anaphylactic reactions may occur in some patients, presenting unique clinical management challenges (6).

In this study, we report a series of four patients who experienced infusion-related hypersensitivity reactions following cadonilimab administration across different tumor types (**Table 1**). We systematically summarize the clinical presentations, management strategies, and outcomes of these cases. Additionally, we provide a comprehensive literature review of cadonilimab’s clinical development, encompassing published clinical trials, case reports, and real-world studies to offer further context on the safety profile and clinical efficacy of this novel bispecific antibody.

**Table 1:** Summary of cadonilimab-induced hypersensitivity reactions in different solid tumors.

Case	Age/Sex	Primary diagnosis	Cadonilimab use	Reaction manifestation	Management	Outcome
1	77/Male	Right lung cancer	Monotherapy after chemotherapy	Dizziness, chest tightness	Infusion stopped, antiallergic therapy	Recovered
2	68/Female	Metastatic gastric cancer	Combined with apatinib	Rash, pruritus, hypotension	Corticosteroid + antihistamine	Discharged
3	66/Female	Cervical cancer with lung metastases	Monotherapy after prior ICI failure	Chest tightness, dyspnea, abdominal pain, hypotension	Methylprednisolone + promethazine	Recovered
4	50/Female	Metastatic cervical adenocarcinoma	Combined with chemotherapy	Anemia, hypoalbuminemia, generalized weakness	Supportive care	Home-based care

## Case presentations

### Case 1

A 77-year-old male was diagnosed with right lung cancer (cT3NxM1) in November 2021. He underwent two courses of six-cycle chemotherapy with stable disease (SD) initially and progressive disease (PD) subsequently. In November 2022, cadonilimab was introduced every three weeks. After receiving three cycles, the patient reported intolerance and switched to serplulimab therapy. Additionally, radioactive seed implantation was performed once. On June 26, 2023, during the cadonilimab infusion period, the patient experienced dizziness and chest tightness suggestive of an infusion reaction. The infusion was discontinued, antiallergic management was initiated, and the patient recovered promptly.

### Case 2

**Figure 1:**
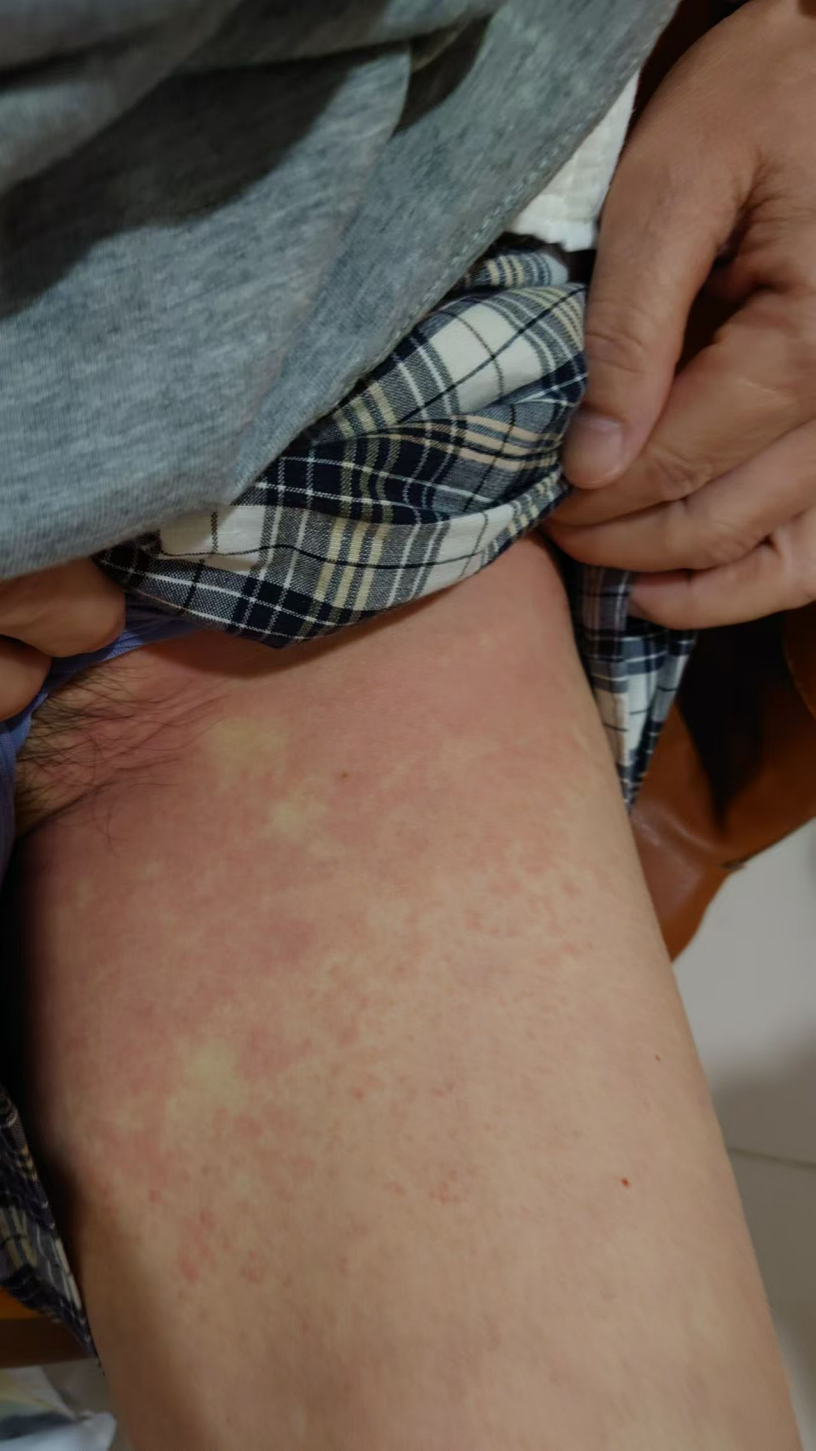
Rash and pruritus observed in a patient during cadonilimab infusion.

**Figure 2:**
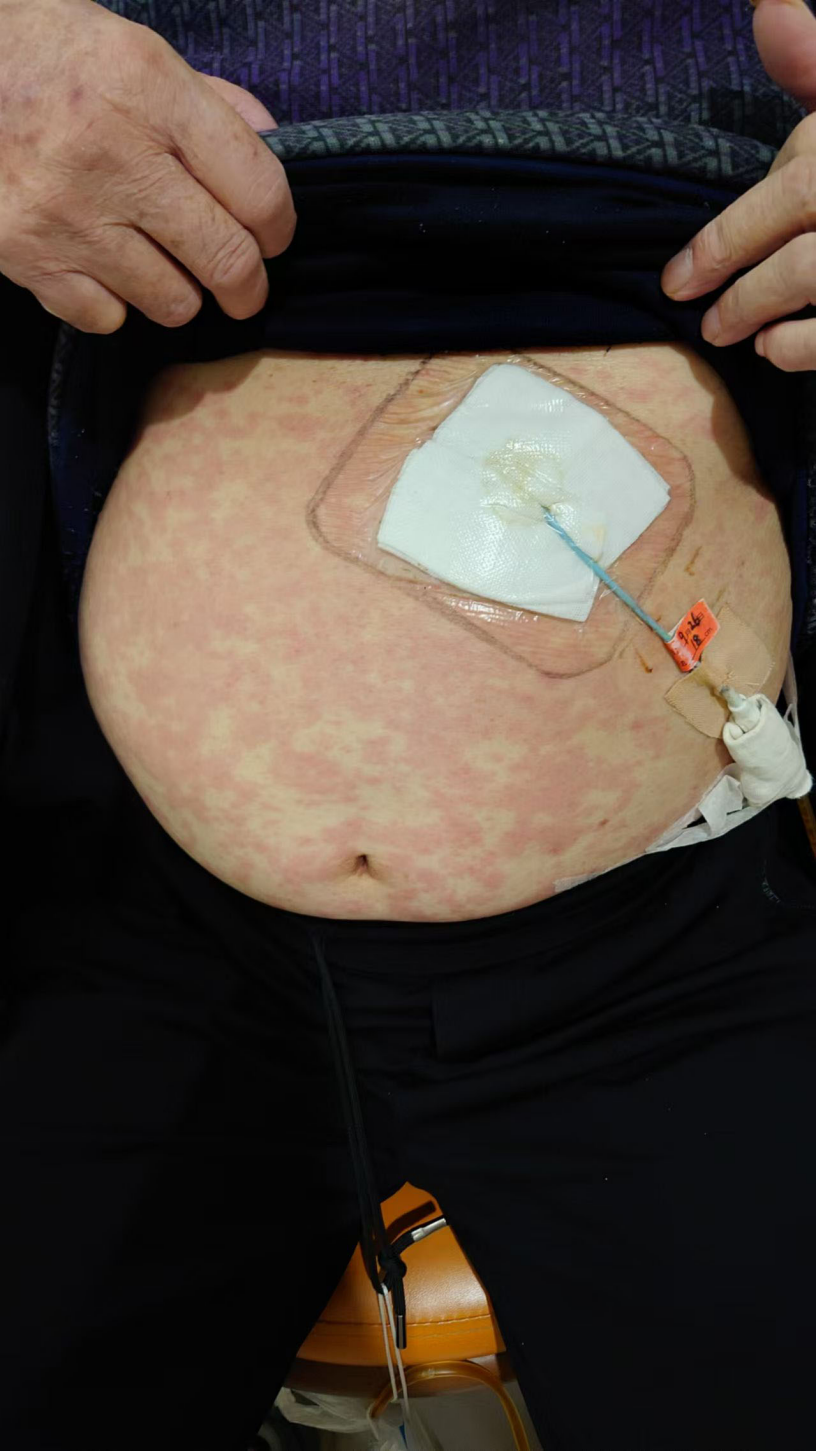
Skin reaction with erythema and pruritus at the infusion site during cadonilimab treatment.

A 68-year-old female presented with gastric cardia adenocarcinoma with extensive metastases to the bone, liver, lymph nodes, and pelvic muscles. Initial treatment included six cycles of oxaliplatin (150 mg on day 1), S-1 (40 mg), and nivolumab (340 mg on day 1 every 3 weeks [d1 q3w]), resulting in partial response (PR). Subsequent progression occurred in June 2023 after 11 months of progression-free survival. On July 28, 2023, she initiated capecitabine (0.5 g once daily [qd] + 1 g once daily [qd] on days 1–14) combined with cadonilimab (375 mg on day 1 every 3 weeks [d1 q3w]) and apatinib. During the second cycle on September 11, 2023, she developed diffuse rash, pruritus, and hypotension (**Figures 1**, **2**), with a significant drop in blood pressure. Immediate management included dexamethasone 10 mg intravenous (IV), methylprednisolone 40 mg intravenous (IV), intravenous fluids, and supportive therapy. Her condition stabilized, and she was discharged in stable condition.

### Case 3

A 66-year-old female with cervical squamous carcinoma and lung metastases had previously received multiple treatment regimens, including paclitaxel, carboplatin, pembrolizumab, and anlotinib. Following disease progression, cadonilimab 250 mg was initiated. On September 26, 2023, during the infusion, she developed chest tightness, dyspnea, abdominal pain, and hypotension. Emergency management included methylprednisolone 40 mg intravenous (IV), promethazine 12.5 mg intramuscular (IM), dexamethasone 5 mg IV, and fluid resuscitation. The patient recovered promptly and remained clinically stable thereafter.

### Case 4

A female patient diagnosed with metastatic cervical adenocarcinoma in 2024 received on March 22, 2025 paclitaxel 240 mg IV over 1 h (premedication: dexamethasone 10 mg IV, diphenhydramine 25 mg IV, famotidine 50 mg IV), followed by carboplatin 550 mg IV over 30 min and cadonilimab 480 mg IV over 1 h. Approximately five minutes into the cadonilimab infusion, she developed facial and generalized erythema with pruritus and hypotension (blood pressure fell from 120/80 mmHg to 85/55 mmHg); post-infusion labs revealed anemia (Hb 61 g/L), elevated inflammatory markers, hypoalbuminemia, and systemic weakness. Rescue treatment with dexamethasone 10 mg IV, methylprednisolone 40 mg IV, promethazine 25 mg IM and intravenous fluids led to rapid symptom resolution. Having tolerated three prior cycles of paclitaxel-based chemotherapy with identical premedication without hypersensitivity—and with this reaction occurring exclusively during cadonilimab infusion—cadonilimab is strongly implicated as the likely trigger. The patient stabilized after treatment but opted for home-based care per personal preference.

## Literature review

As of June 1, 2025, a search using the keyword “Cadonilimab[Title]” on PubMed yielded 43 publications. Based on this, we systematically reviewed and summarized all available clinical trials and case reports related to cadonilimab (AK104), organizing the data into a comprehensive tabular format (**Table 2**, **Table 3**).

**Table 2:** Case reports.

No.	Title	Indication	Type	Key finding
1	A surprising complete response to cadonilimab in a primary metastatic cervical cancer	Cervical Cancer	Case Report	CR for 10 months
2	Advanced cervix cancer patient with chemotherapy-induced grade IV myelosuppression achieved complete remission with cadonilimab	Cervical Cancer	Case Report	Remission after chemotherapy intolerance
3	Multiple primary tumors patient developed microsatellite stable gastric cancer after cadonilimab treatment for liver cancer	Gastric + Liver Cancer	Case Report	Multiple primaries benefited
4	Combination of cadonilimab and apatinib as salvage therapy in MSI-H gastric cancer	MSI-H Gastric Cancer	Case Report	Immunotherapy + targeted therapy benefited
5	Therapeutic response of cadonilimab plus chemotherapy in STK11-mutant NSCLC	STK11-mutated NSCLC	Case Report	Response in frontline treatment
6	Efficacy of cadonilimab and anlotinib in drug-resistant pulmonary LCNEC	LCNEC	Case Report	Immuno-targeted combo responded
7	Cadonilimab plus chemotherapy in superaged gastric cancer patient	Superaged Gastric Cancer	Case Report	85 y/o patient sustained remission
8	Cadonilimab-related toxic epidermal necrolysis-like reactions successfully treated with supplemental Adalimumab	Liver Cancer	Case Report	Severe TEN managed with Adalimumab
9	Patients with positive HER-2 amplification advanced gastroesophageal junction cancer achieved complete response with combined chemotherapy of AK104/cadonilimab	HER2+ GEJ Cancer	Case Report	CR in HER2+ patient

**Table 3:** Clinical studies.

No.	Title	Indication	Study type	Key result
1	Safety and antitumour activity of cadonilimab (COMPASSION-03)	Advanced Solid Tumors	Phase 1b/2	ORR: Cervical 32%, ESCC 18%, HCC 17%
2	Efficacy and safety of cadonilimab + lenvatinib (COMPASSION-08)	HCC	Phase Ib/II	ORR 35%, PFS 8.6-9.8mo, OS up to 27mo
3	Cadonilimab Combined with Chemotherapy ± Bevacizumab (COMPASSION-13)	Cervical Cancer	Phase II	ORR 66-92%
4	Efficacy of cadonilimab in previously treated R/M NPC (COMPASSION-06)	Nasopharyngeal Cancer	Phase II	ORR 26%, 12mo OS 79%
5	Cadonilimab + platinum chemo ± bevacizumab (COMPASSION-16)	Cervical Cancer	Phase III	PFS 12.7 vs 8.1mo; OS improved
6	Cadonilimab with chemo in HER2-negative GEJ (COMPASSION-04)	Gastric/GEJ Cancer	Phase 1b/2	ORR 52%, mOS 17mo
7	First-line cadonilimab + chemotherapy (Nature Medicine)	Gastric/GEJ Cancer	Phase III	Main registrational trial
8	Nab-paclitaxel + cadonilimab 2nd-line gastric	Gastric Cancer	Phase II	Immune rechallenge exploration
9	Cadonilimab + SOX neoadjuvant	Gastric Cancer	Phase II	SOX-based neoadjuvant
10	Neoadjuvant cadonilimab + FLOT	Gastric Cancer	Phase II	pCR 21%, MPR 45%
11	Cadonilimab + chemo for advanced/recurrent endometrial cancer	Endometrial Cancer	Phase II	45 planned
12	Short-course RT + chemo + cadonilimab in rectal cancer	Rectal Cancer	Phase II	50 planned
13	AK104-202 study in NSCLC	NSCLC	Phase Ib/II	ORR 10%, OS 19.6mo
14	First-in-human study (COMPASSION-01)	Multiple Tumors	Phase Ia/Ib	ORR 13%
15	Cadonilimab + Anlotinib in NSCLC	NSCLC	Phase Ib/II	ORR 51-60%
16	Cadonilimab Phase II in ES-SCLC	SCLC	Phase II	Preliminary safety
17	Real-world: cadonilimab + TKI in uHCC	HCC	Real-world	ORR 38%, OS 13.7mo
18	Real-world: cadonilimab + lenvatinib in HCC	HCC	Real-world	ORR 37%, PFS 8.1mo
19	Cost-effectiveness analysis (gastric cancer)	Gastric Cancer	Health Economics	ICER: $67,378/QALY
20	Cost-effectiveness analysis (cervical cancer)	Cervical Cancer	Health Economics	ICER: $70,220-75,944/QALY
21	Cost-effectiveness (COMPASSION-16 cervical cancer)	Cervical Cancer	Health Economics	WTP threshold analysis

Cadonilimab, a first-in-class bispecific antibody targeting both programmed death-1 (PD-1) and cytotoxic T-lymphocyte-associated antigen-4 (CTLA-4), has undergone extensive clinical development in a wide range of solid tumors, demonstrating broad-spectrum antitumor activity and manageable safety profiles (7–9).

Currently, based on published clinical studies and case reports, cadonilimab has been explored across multiple tumor types, including gastric/gastroesophageal junction (GEJ) cancer, cervical cancer, hepatocellular carcinoma (HCC), non-small cell lung cancer (NSCLC), small cell lung cancer (SCLC), large cell neuroendocrine carcinoma (LCNEC), nasopharyngeal carcinoma, endometrial cancer, and rectal cancer. The available evidence spans phase I to phase III clinical trials, real-world studies, and health economics analyses (data source: systematic literature integration, N=30) (10–13).

In registrational trials, the phase III COMPASSION-16 study established the survival benefit of cadonilimab combined with chemotherapy (± bevacizumab) for patients with recurrent or metastatic cervical cancer, with a progression-free survival (PFS) improvement to 12.7 months (HR = 0.62; NCT04982237) (12, 14). In HER2-negative advanced gastric cancer, the phase 1b/2 COMPASSION-04 trial demonstrated an objective response rate (ORR) of 52.1% and a median overall survival (OS) of 17.5 months (CTR20182027). Additionally, in advanced hepatocellular carcinoma, the phase Ib/II COMPASSION-08 trial of cadonilimab combined with lenvatinib achieved an ORR of 35% and a median PFS of 8.6–9.8 months (15).

Real-world evidence and case reports further expanded the application spectrum of cadonilimab in various special patient subgroups (10). Successful treatment outcomes have been reported in patients with MSI-H gastric cancer, HER2-amplified gastric cancer, STK11-mutant NSCLC, PD-1-resistant nasopharyngeal carcinoma, multiple primary malignancies, and super-aged populations. Furthermore, several severe immune-related adverse events (irAEs), such as toxic epidermal necrolysis (TEN-like reactions), immune hepatitis, and immune pneumonitis, have also been documented, and most were successfully managed with corticosteroids and biological agents (13).

In summary, cadonilimab demonstrates promising potential for multi-indication development through combination strategies with chemotherapy and targeted agents. Particularly, cadonilimab offers new therapeutic opportunities in traditionally immunotherapy-resistant populations (e.g., HER2-positive, STK11-mutated, and microsatellite stable gastric cancer). Its bispecific structure design may also reduce the incidence of severe immune-related toxicity compared to conventional PD-1 plus CTLA-4 combinations, positioning cadonilimab as a next-generation platform in immune-oncology development.

## Discussion

Cadonilimab, a novel PD-1/CTLA-4 bispecific antibody, has demonstrated promising antitumor efficacy across multiple solid tumors, including cervical cancer, gastric cancer, hepatocellular carcinoma, and non-small cell lung cancer. However, as with other immune checkpoint inhibitors (ICIs), cadonilimab carries the risk of immune-related adverse events (irAEs), including rare but potentially life-threatening hypersensitivity or infusion-related reactions. Owing to its bispecific structure, cadonilimab may theoretically exhibit more complex immune activation and hypersensitivity profiles compared to monospecific PD-1 or CTLA-4 inhibitors (5, 16, 17).

Furthermore, although we initially considered the delayed onset of infusion reactions after the second or third dose to be inconsistent with a true allergic mechanism, this interpretation may be oversimplified. Indeed, a true IgE-mediated or T-cell–mediated allergic response can manifest with rapid symptoms after the second or subsequent exposures, as prior sensitization may prime the adaptive immune response. Therefore, the occurrence of rapid-onset hypersensitivity reactions after multiple infusions of cadonilimab in our series could still be compatible with a true allergic mechanism, and warrants further immunological investigation.

In addition, an alternative mechanism involving MRGPRX2-mediated mast cell activation has been proposed for monoclonal antibody–related reactions. This pathway can trigger non–IgE-mediated anaphylactoid responses; however, such reactions are typically more pronounced during the first administration. Since our cases experienced hypersensitivity reactions after several treatment cycles, the role of MRGPRX2 alone may be less likely (18–20). Future *in vitro* testing of cadonilimab on mast cell degranulation models could help to further clarify its involvement.

Finally, it is important to note that cadonilimab has an Fc-null engineered backbone, which reduces Fc receptor–dependent immune activation and potentially lowers certain immune-related adverse events (16). Nevertheless, this modification does not eliminate the risk of all hypersensitivity or immune-mediated reactions. Therefore, these explanations remain speculative, and caution is warranted in interpreting the comparatively favorable safety profile of cadonilimab without further immunologic confirmation. The occurrence of severe reactions in our patients suggests that other pathways—beyond Fc engagement—may contribute to these infusion-related events, highlighting the need for close monitoring and mechanistic research.

Although paclitaxel is a recognized cause of infusion-related hypersensitivity, several lines of evidence in this patient argue against it being the culprit. First, the paclitaxel infusion (preceded by standard dexamethasone, diphenhydramine, and famotidine premedication) was completed uneventfully, and the acute reaction began only after cadonilimab infusion commenced. Second, the patient had previously tolerated three cycles of paclitaxel-based chemotherapy with identical premedication without any hypersensitivity. Third, the rapid onset of rash, pruritus, and hypotension—within 5 minutes of starting cadonilimab—is more consistent with reactions reported for PD-1/CTLA-4 bispecific antibodies than with classical paclitaxel reactions (21). Taken together, these observations strongly implicate cadonilimab rather than paclitaxel as the primary trigger in Case 4.

The clinical spectrum of hypersensitivity reactions in our patients was heterogeneous, ranging from mild cutaneous manifestations (rash, pruritus) to more severe systemic symptoms, including hypotension, dyspnea, and gastrointestinal discomfort (**Table 4**). Notably, one patient experienced hypotension without loss of consciousness, while others displayed combinations of multisystem involvement. This variability underscores the importance of early recognition and prompt intervention. In all cases, immediate interruption of infusion and initiation of anti-allergic therapy successfully stabilized patients, preventing further clinical deterioration (22, 23).

**Table 4:** Premedication, infusion adjustments, and outcomes of cadonilimab rechallenge in hypersensitivity cases.

Case	Premedication	Infusion adjustment	Reaction manifestation	Outcome	Treatment cycle
1	Dexamethasone 10 mg IV, diphenhydramine 25 mg IV, famotidine 50 mg IV (30 min before infusion)	Cadonilimab 250 mg in 250 mL saline, infused over 2 h (instead of 1 h)	No rash or hypotension; completed four further cycles uneventfully	No hypersensitivity events	Cycle 1
2	Methylprednisolone 40 mg IV, chlorpheniramine 10 mg IV, famotidine 50 mg IV	Stepwise infusion: 10% dose over 30 min → observe 30 min → remaining 90% over 90 min	Mild flushing during initial 10% infusion (resolved with extra antihistamine); full dose completed without further events	No further events	Cycle 2
4	Dexamethasone 10 mg IV, diphenhydramine 25 mg IV, famotidine 50 mg IV (1 h before infusion)	Extended infusion to 2 h, with first 15 min at 5 mg/h	Only transient pruritus; no hypotension or rash	Symptoms resolved with treatment	Cycle 1

Importantly, three patients in our series were able to resume cadonilimab therapy following the initial hypersensitivity event. Reintroduction was achieved either through reduced infusion rates, corticosteroid and antihistamine premedication, or modified dosing schedules. This experience aligns with previously reported desensitization and rechallenge strategies for other ICIs, suggesting that hypersensitivity events do not invariably necessitate permanent discontinuation of therapy, particularly when clinical benefit remains substantial.

In addition to the infusion-related hypersensitivity reactions observed in our reported cases, severe immune-related adverse events (irAEs) associated with cadonilimab have also been documented in the literature (24). For example, a recently published case reported toxic epidermal necrolysis (TEN)-like reactions induced by cadonilimab in a hepatocellular carcinoma patient receiving combination therapy, which were successfully managed with supplemental adalimumab. This case highlights that although cadonilimab’s Fc-null structure is designed to reduce immune activation, significant irAEs can still occur and warrant careful monitoring and prompt intervention. Our case series, together with these reports, suggests that both immediate-type hypersensitivity reactions and delayed immune-related toxicities should be considered as part of the safety profile of cadonilimab. Further pharmacovigilance and mechanistic studies are needed to elucidate risk factors and optimal management strategies for these events.

As cadonilimab continues to expand its clinical indications across diverse tumor types and treatment settings, hypersensitivity reactions warrant heightened clinical awareness. Proactive risk stratification, early symptom recognition, and well-prepared management protocols are critical to ensuring both patient safety and therapeutic continuity (3, 25). Moreover, larger prospective studies are needed to elucidate the underlying immunopathogenesis of cadonilimab-induced hypersensitivity and to establish evidence-based desensitization or prophylactic algorithms for high-risk patients.

## Conclusion

Cadonilimab-induced hypersensitivity reactions present clinical challenges requiring multidisciplinary management. With increasing clinical application, heightened vigilance, prompt intervention, and individualized rechallenge strategies may allow continued benefit from immunotherapy in select patients.

## Data Availability

The original contributions presented in the study are included in the article/supplementary material. Further inquiries can be directed to the corresponding author.
